# Making Childhood Obesity a Priority: A Qualitative Study of Healthcare Professionals' Perspectives on Facilitating Communication and Improving Treatment

**DOI:** 10.3389/fpubh.2021.652491

**Published:** 2021-07-15

**Authors:** Costela Lacrimioara Serban, Salomeia Putnoky, Anna Ek, Karin Eli, Paulina Nowicka, Adela Chirita-Emandi

**Affiliations:** ^1^Functional Sciences Department, “Victor Babes” University of Medicine and Pharmacy Timişoara, Timişoara, Romania; ^2^Microbiology Department, Centre for Studies in Preventive Medicine, “Victor Babes” University of Medicine and Pharmacy Timisoara, Timisoara, Romania; ^3^Division of Pediatrics, Department of Clinical Science, Intervention and Technology, Karolinska Institutet, Stockholm, Sweden; ^4^Division of Health Sciences, Warwick Medical School, University of Warwick, Coventry, United Kingdom; ^5^School of Anthropology and Museum Ethnography, University of Oxford, Oxford, United Kingdom; ^6^Department of Food Studies, Nutrition, and Dietetics, Uppsala University, Uppsala, Sweden; ^7^Department of Microscopic Morphology Genetics Discipline, Center of Genomic Medicine, Regional Center of Medical Genetics Timis, “Victor Babes” University of Medicine and Pharmacy Timisoara, Timisoara, Romania; ^8^Regional Center of Medical Genetics, “Louis Turcanu” Clinical Emergency Hospital for Children, Timişoara, Romania

**Keywords:** children, family, overweight, obesity, parents, stigma, STOP project, thematic analysis and constructs

## Abstract

In Romania, one in four children has excess weight. Because childhood obesity is a sensitive topic, many healthcare professionals find it difficult to discuss children's excess weight with parents. This study aims to identify barriers and facilitators in childhood obesity-related communication, as perceived by healthcare professionals in Romania. As part of the STOP project, healthcare professionals (family physicians, pediatricians, and dieticians) who treat children with excess weight were invited to a telephone interview. The semi-structured questions were translated from a questionnaire previously used at the Swedish study site of the STOP project. Interviews were transcribed and then used for thematic analysis. Fifteen doctors and three dieticians (16 females and 2 males), with average 18.2 ± 10.1 years of experience, were interviewed. Four main themes were identified. Professionals reported that when children began experiencing obesity-related stigma or comorbidities, this became the tipping point of weight excess, where parents felt motivated to begin treatment. Barriers in communication were part of several layers of distrust, recognized as tension between professionals and caregivers due to conflicting beliefs about excess weight, as well as lack of trust in medical studies. Most respondents felt confident using models of good practice, consisting of a gentle approach and patient-centered care. Nonetheless, professionals noted systemic barriers due to a referral system and allocation of clinical time that hinder obesity treatment. They suggested that lack of specialized centers and inadequate education of healthcare professional conveys the system does not prioritize obesity treatment and prevention. The interviewed Romanian doctors and dieticians identified patient-centered care as key to treating children with obesity and building trust with their caregivers. However

their efforts are hindered by healthcare system barriers, including the lack of specialized centers, training, and a referral system. The findings therefore suggest that, to improve childhood obesity prevention and treatment, systemic barriers should be addressed.

**Trial Registration:** ClinicalTrials.gov, NCT03800823; 11 Jan 2019.

## Introduction

Excess weight in children is prevalent worldwide. Although plateauing trends were observed in many high-income countries in Europe, in medium and low-income countries childhood obesity prevalence has increased in the last decade (1). In Romania, almost one in four children has overweight or obesity, as shown in a pooled analysis of more than 25,000 school age Romanian children (2) and the European Childhood Obesity Surveillance Initiative (COSI) (3).

Childhood obesity is recognized as a significant concern in the 2014–2020 Romanian National Health Strategy (HG, No.1028/18.11.2014) and efforts have been made to implement policies supporting healthy food and activities in the school setting. Additionally, media campaigns were coordinated to promote a healthy lifestyle. The Health Ministry's strategy did not formally name the healthcare professionals who should treat childhood obesity; however, family physicians, general, and specialist pediatricians (endocrinologists and specialists in diabetes and metabolic diseases), together with dieticians, are key to childhood obesity treatment.

Communicating with children and families about excess weight represents a crucial clinical skill for healthcare professionals in the prevention and management of childhood obesity (4). Focusing on obesity is difficult for several reasons, including time constraints and a concern about how the message is perceived (5). Previous studies have shown that many healthcare providers hold negative attitudes toward people with obesity (6). Such stigma can lead to reduced quality of care for people with obesity despite the best intentions of healthcare professionals (6, 7). A joint international consensus statement for ending weight stigma was recently published, in an attempt to raise awareness of the negative consequences of weight stigma among a broad group of stakeholders, including healthcare providers, researchers, the media, policymakers, and patients (8). However, even when healthcare professionals do not endorse weight stigma, the social stigmatizing of obesity may affect the quality of care. Healthcare professionals are often avoiding discussing obesity with patients and their families, for fear of offending them and losing their trust (9).

The success of childhood obesity treatment depends on improving healthcare professionals' education, attitudes, and practices related to communication about obesity, as well as coordinated efforts to reduce weight stigma on the community level (10, 11). A recent meta-analysis has shown that numerous studies explored weight stigma and healthcare communication in North America and Western Europe (12). However, similar studies have not been performed in Central/Eastern Europe, and no study has investigated how Romanian healthcare professionals perceive communication about excess weight. Overall, research on childhood obesity treatment in Central/Eastern Europe is limited, as shown by a recently updated Cochrane review (13).

This study presents the first analysis of barriers and facilitators Romanian healthcare professionals face when communicating with and treating families of children with excess weight. Through this, we aim to develop an in-depth understanding of the locally-specific conditions healthcare professionals face, and thereby contribute to the improvement of childhood obesity treatment in Romania.

## Method

### Participants

Healthcare professionals (family physicians, pediatricians, and dieticians) who treat children with excess weight were recruited for the study. Doctors were identified through a public registry of 242 family physicians and 54 pediatricians. A formal list of pediatric dieticians was not available, therefore, a sample of the author's professional contacts were invited to participate. All healthcare professionals were recruited in Timisoara, a metropolitan area in western Romania. Timisoara's metropolitan area is home to almost half a million inhabitants and is one of Romania's economic hubs, as shown by gross domestic product per region (14). Potential participants were initially invited by email. The invitation emails explained the purpose and procedure of the current study. Interviews were scheduled with those who responded within a 3-week timeframe. The number of participants has met the criteria for data saturation established by Guest et al. (15).

### Interview Guide

The present study is part of the EU funded project “Science and Technology in childhood Obesity Policy” (STOP) (Grant Agreement No. 774548). We used the questionnaire and interview guide developed by Sjunnestrand et al. (9) at the STOP project's Swedish study site. The semi-structured interview questions aimed to capture respondents' experiences of communicating with parents about their children's excess weight and to identify the perceived barriers and facilitators for inception of obesity treatment. The Swedish guide was translated into English and then to Romanian with minor reformulations and cultural adaptations.

### Interviews

The interviews were conducted one-on-one by a female researcher (SP), with professional experience in medicine and dietetics (MD, Ph.D.) and teaching experience of 26 years. She asked all respondents the same set of core questions, as well as individualized follow-up questions based on the responses, using think-aloud and verbal probing techniques (16). Both techniques provide a better understanding of the cognitive processes induced by the questions, enabling participants to express their own thought processes and raise additional issues. All interviews were conducted via telephone. The interviews were audio recorded and then transcribed verbatim by members of the research team (IJS, CLS, MB, AD). Field notes were made after the interviews.

### Thematic Analysis

The interview transcripts were analyzed in Romanian, using thematic analysis. The transcribed interviews were read, re-read and then coded by ACE and CLS, using an inductive approach (data driven), rather than being limited to a pre-existing coding frame stipulated by the initial hypothesis (17). Thus, identified themes related to the responses rather than the specific interview questions. In using an inductive approach, we did not employ a predetermined theoretical framework; however, the coders were particularly interested in data that addressed barriers and facilitators to communication. All codes were defined in a codebook following a template (18). ACE and CLS had several meetings to follow the progress of analysis and to discuss the coding. Themes and subthemes were developed from the codes, following wider team discussions between ACE, CLS, KE and PN, in which a few disagreements were resolved and consensus achieved. Relevant quotes for the subthemes were translated from Romanian to English by ACE and CLS.

The study received ethics approval from the Scientific Research Ethics Committee Board of the “Victor Babes” University of Medicine and Pharmacy Timisoara (no.06/02.03.2020). Participant confidentiality is maintained throughout the manuscript. Each quote is labeled to indicate the position of each respondent and the interview number, as follows: (1) gender (F -female/M-male), (2) number within group, (3) group: primary care (PC) represented by family physicians and general pediatricians; secondary care (SC) represented by specialist pediatricians (gastroenterology, cardiology, nephrology, orthopedics, genetics); tertiary care (TC) represented by pediatric specialists designated for obesity healthcare (endocrinology, diabetes) and dieticians; (4) years of experience. The COnsolidated criteria for REporting Qualitative research (COREQ) checklist (Supplement 1) was used in creating the report.

## Results

Initially, 21 participants agreed to be interviewed; however, three of them were not available to schedule an interview in the proposed timeframe due to time constraints (2 persons) and health issues (1 person). The final analysis included interviews with 18 respondents, all of whom verbally expressed informed consent and provided answers to all questions.

**Figure 1:**
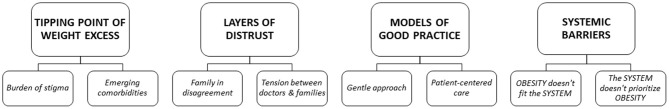
Main themes and sub-themes identified.

In total, 18 physicians and dieticians (16 females and 2 males), with average 18.2 ± 10.1 years of experience (range 5–35 years), were included in the analysis. Physicians in primary care (2 family physicians, 6 general pediatricians) had on average 17.75 ± 10.4 years of experience. Physicians in secondary care (5 specialist pediatricians in cardiology, nephrology, genetics, orthopedics, and gastro-enterology) had on average 21.2 ± 9.0 years of experience. Healthcare professionals in tertiary care (one pediatric specialist in endocrinology, one specialist in diabetes care and 3 dieticians) had on average 15.2 ± 12.0 years of experience. The average interview time was 26 min (range 14–49 min). Four main themes and eight sub-themes were identified (presented in Figure 1).

## Tipping Point of Weight Excess

The first theme, “Tipping point of weight excess,” captured the triggers that motivated families to seek obesity treatment. The respondents noted that families did not perceive excess weight as a problem until a tipping point was reached and obesity became a priority. The determining moment for families/individuals was associated with the burden of stigma (first subtheme) and emerging comorbidities (second subtheme).

### Burden of Stigma

Although some physicians described excess weight discussions as routine, the majority felt that children's excess weight was a sensitive subject for caregivers. This was especially the case for physicians who were the first to raise the topic. On the contrary, dieticians did not consider excess weight as a particularly sensitive topic, as they saw families after they had been self-referred or referred by a physician, and were therefore already prepared for discussions of excess weight.

Most healthcare professionals deliberately used non-offensive words in raising the issue of childhood excess weight:

“*I try to be very careful, I don't use the words obesity or fat, I may say that he/she weighs a few extra kilograms and that he/she should take some measures to prevent worse situations, (…) I try an approach with kid gloves, more gentle, to see how parents react”* (F5PC30).

Practitioners were aware of the effects obesity stigma can have on families, and actively tried to avoid connotations of stigma when speaking to patients and parents:

“*trying to keep a balance between avoiding stigma for the patient and explaining that excess weight is a problem,. it is not about aesthetics or trying to fit into a body ideal, it is about the health consequences of obesity (.) To stigmatize is not in our interest, as we try to obtain the patient's compliance”* (F3TC33).

However, children and families often encountered stigma before meeting the physician. Parent and family awareness was sometimes triggered by a shocking event in a social context or a school setting, such as an incident of bullying. For example, one physician said:

“*I think there is a context, in school, or somewhere else, a situation that triggers this, and all of a sudden the parent understands -.Ooo! my child is…- even though in the last years the child was chubby and nothing happened”(F2TC6)*.

Stigma burden in the school setting was reported in almost all interviews. “*Older children, girls, are generally bothered by being overweight because they have problems at school. They are bullied (…)”* (F2TC6). In school, stigmatization by peers was worse in sports contexts, where physical capabilities and endurance were evaluated: “*the child cannot integrate in the sports class because he cannot cope and his peers laugh or the teacher points out the difference in performance”* (F2TC6). According to most respondents, stigmatization at school was a key tipping point that urged children and parents to seek help. For example, one physician spoke about a patient who said “*that she really wants to lose weight because she gets tired too easily and is ashamed during sport classes and in school”* (F4PC9). Another respondent also mentioned: “*When children are socially discriminated against, the parents are starting to feel that something is wrong and want to make a change” (F1TC12)*.

### Emerging Comorbidities

Respondents reported that some families approached healthcare practitioners only when the child's excess weight became moderate or severe: “*Never with small excess (weight), always when the excess outruns some threshold, when they feel it's beyond their control (.) in most, when the excess is moderate to severe”* (F5TC23). In many cases, families contacted physicians due to comorbidities of excess weight rather than due to the excess weight itself:

“*Most do not contact us due to being overweight, rather (.) for other (*causes*). asthma, hypertension or diabetes or something else, but do not come to the hospital due to obesity, do not come to ask for help in losing weight”* (F7PC15).

In preschoolers, orthopedic complaints, gastroenterological issues, recurrent respiratory infections, and sleep disturbance were mentioned as drivers to seek medical care. As one respondent described:

“*A very high percentage asks for help when other health problems arise, that can be felt. That is, concretely, the child feels ill, the child does not breathe well, the child snores, does not sleep at night, does not rest”* (F2PC7).

Another respondent noted:

“*They decide [to seek help] when their children have an orthopedic problem, they have joint pain, pain in their lower limbs or back or when they have respiratory infections, (…) more frequently compared to their friends”* (F1PC30).

In adolescent patients, comorbidities usually found in adults, such as arterial hypertension, metabolic syndrome, and endocrine dysfunctions were reported.

Physicians reported that, even after seeking help for a child's obesity-related illness, parents still did not recognize obesity as the underlying issue, and sometimes did not understand that excess weight could contribute to the condition for which they seek treatment:

“*Most of them come for gastroenterology consultation, due to high levels of transaminase or irritable bowel syndrome or dyspepsia, which are clearly connected with obesity. But they (the caregivers) do not see obesity as a problem, they only see these symptoms and focus on them”* (F4SC12).

Nonetheless, physicians explained that, even when parents did not recognize obesity as a problem, they used the child's referral for another condition to initiate a conversation about obesity: “*They come for other pathologies - breathing problems, most often, and then, we take the opportunity to talk about obesity”* (F2PC7).

## Layers of Distrust

The second overarching theme identified in the interviews was *layers of distrust*. This theme captures disagreements about excess weight and food practices in the family, alongside an intrafamilal “blame game.” It also captures conflicting views of obesity, as expressed by families and healthcare professionals, and families' lack of trust in the medical system. Two subthemes were identified: family in disagreement and tension between doctors and families.

### Family in Disagreement

Respondents noted that family members often disagreed about the meaning and severity of excess weight, and that this had implications for children's treatment. As one respondent said,“*(.) most unsuccessful outcomes are due to family disagreeing views”* (F3PC10). According to the respondents, the presence of both parents in the clinical consultation increased the level of agreement and strengthened the chances of treatment success. When children were accompanied by one caregiver, usually the mother, she disclosed that other member(s) of the family did not agree with seeking help for the child's excess weight, usually because they felt excess weight was not urgently problematic. One respondent explained that “*[m]ost of the time both parents agree that there is a problem, (but) the way they respond to the problems is different. At least one of them tends to neglect or minimize the importance of this problem*” (M2SC18).

When children were accompanied by only one caregiver, absent family members were frequently blamed for the child's obesity: “*(.) the guilt is usually assigned to grandparents, or someone else than the person that accompanies the child. (.) Others are (accused of) secretly giving chocolate, snacks and potato chips*” (F7PC15). When possible, a more complete picture of the familial situation was ascertained by conducting separate discussions with different caregivers involved in the child's care: “*things are not exactly as the parents present them. When we talk to grandparents, we learn that the parents are also buying and eating unhealthy foods and then things are more complicated”* (F3TC33).

### Tension Between Doctors and Families

The gap between healthcare professionals' and families' understandings of childhood excess weight was another source of tension. Respondents said that communication barriers occurred more often in meetings with low-income families or with grandparents. Among poorer and older family members, the respondents explained, children's excess weight was associated with higher social status, health and beauty. For example, one respondent said a grandmother confronted her, saying “*[y]ou (the doctor) cannot tell me that something is wrong with my grandchild, that he is too fat. He is a child that eats well”* (F1SC16). Another respondent described remarks such as: “*- Ma'am, this is not a problem, I mean, I am also white, fat and beautiful”;* this practitioner explained that “*[t]his creates a communication barrier, that I felt that I will have no success, no matter how I approach it”* (F2PC7).

Respondents noted that families often refused to participate in studies concerning excess weight. A recurrent opinion was that parents worried their children would be treated as “guinea pigs” in clinical studies: “*I don't know what it's like in other countries, but in Romania they don't like to be studied. They fear they will become a guinea pig. I think it's a matter of perception and I don't know how it could be changed”* (F4SC12). Respondents suggested that families' concerns were driven by not understanding the benefits of research and the belief that no feedback will be provided, as well as low parental education level, the socio-cultural valuation of children's excess weight, and the additional effort that research participation involves: “*The complexity of these studies and the fact that we ask parents and children make an extra effort, efforts that I do not think they are willing to make (…) Somewhere here is the barrier”* (M2SC18). A potentially key reason for refusal to participate in studies was lack of trust in the healthcare system. One respondent reported that she encountered similar refusal to participate in studies on diabetes and obesity across Romania:

“*Because there is a general distrust in the medical staff in Romania (.). Distrust is also showed by the refusal to vaccinate children (.) We did not experience this 10-12 years ago, instead, recently, for a study regarding diabetes, two years ago it was a mass refusal, and not only in Timisoara but also in other parts of the country. (…) In studies that require blood sampling, interventional studies or studies that include medication, there are already more reluctant. I can't say that these refusals are justified, but unfortunately I found refusals like that … without any a justification”* (F3TC33).

## Models of Good Practice

Most respondents reported they use various models of good practice to ensure appropriate communication, aiming to empower patients and their families to start treatment. This theme consisted of the two subthemes: gentle approach and patient-centered care.

### Gentle Approach

Respondents recognized the crucial role of language in the clinical encounter: “*Words, including gentle words, can have an impact. We must not be brutal in the way we communicate with the families, in any form”* (F4PC9). Many practitioners reported using a probing interview approach to assess caregivers' awareness of excess weight: “*after I have taken care of the acute pathology that brings them to me, I ask questions like what age did the child start to gain weight, and so, I find out if it is a problem for them, because for many it is not a problem”* (F6PC30). Other respondents reported using a direct approach without labeling the child obese. This was usually achieved by highlighting children's deviations from the normal growth curve: “*based on measurements, we show them exactly the situation of their child versus normal growth line for age and gender (.). On a reference chart we can show the family (.) the healthy weight for the height of their children”* (F3TC33).

When approaching families who were not aware of the risks associated with excess weight, respondents described using a gentle approach that included seeking a “*level of awareness, to be sure that they understand what I want to say to them, (.) that I am not talking about aesthetics, rather, thinking about possible risks that they undertake if they continue with increasing weight”* (F5TC23).

Healthcare professionals recognized that trust could be built step-by-step only when a channel of communication had been opened, with recommendations delineated gradually: “*I think, foremost, you have to earn their cooperation and trust and then recommendations can be enacted progressively, but with their consent. First they must understand and then they will accept”* (F5TC23). Respondents also frequently reported focusing on what could be done and achieved, using positive language. For example, one physician describes speaking to families as follows:

“*you have to look at the positive side all the time: your child has a few extra kilograms, but let's look at the good side, he/she is in a growing period and it's much easier to do something now, as the child is much more physically active and then let's see how we can improve in the future (…) and that kind of works. So, I always tell them that it is much easier for the child to lose weight during periods of growth”* (F7PC15).

To improve communication, respondents made efforts to empower patients and families. For example, one respondent said

“*[i]t helps to communicate and listen to their problems actively. Many times, they just need to be listened to. When they feel understood, they have even more confidence; somehow, they feel that they can do it. They often need encouragement and self-confidence that they can make changes”* (F1TC12).

Another respondent said she made sure to praise her patients: “*I praise the child directly. I ask him if some things have improved (…), I ask how he feels, toward maintaining or losing weight”* (F1PC30).

### Patient-Centered Care

Respondents communicated to families that they were on their side: “*I think it matters a lot to be perceived as an ally, as a person who wants what's best for them and not as an extremely authoritarian figure. As an ally who knows what she is talking about and who gives them informed advice, having experience in this field”* (F2PC7). Respondents also said they adapted to the families' concerns by offering personalized advice, taking into account families' wishes and their financial and social needs. For example, a dietician trying to dismiss a family's perception that healthy food was expensive said:

“*I gave them information about the price of foods a child can eat. To prove that it is not so expensive for a child to eat healthy (.) I give them options and examples - Look, instead, he can eat something that is four times cheaper and healthier. I even give them variants of shopping lists, including stores where to* do grocery shopping. *(.) From the moment I explain the cost (of healthy foods), that it's not so expensive, they have less preconceptions”* (F2TC6).

According to the respondents, good practice entailed the successful involvement of the family in providing supportive environment for the child. They encouraged family members to share home cooked meals and act as role models of healthy eating. When grandparents were in charge of cooking for the child, the respondents invited them to the clinic to attend discussions regarding healthy food choices. For example, one respondent noted, “*[i]n a subsequent conversation we invite the grandparents, (.) and then the grandparents have to support the treatment plan by sharing the same meals as the grandchildren”* (F3TC33).

Almost all respondents agreed that children's presence in clinical visits was beneficial, except if the children were too young to join the discussions. However, even if young children were kept busy with other activities during the visit, the respondents thought it was good for them to be present and to hear the discussion, as one physician pointed out “*[t]hey (*the small children*) understand more than we think”* (F4SC12). If the children were older, respondents said that compliance and outcomes were improved if the child was engaged in the treatment plan. Indeed, one respondent noted that “*doctors can sometimes work better with the child than with the parent”* (F7PC15). Some practitioners reported using an age-and gender-specific approach:

“*If the child is small, I do not approach the child, I approach the parent. If the child is a female preadolescent, I try to approach it differently. I tell the parent very clearly that if she does not control her excess weight at this age, she is at risk to have distorted body image perception that might lead to eating disorders in adolescence. In boys, I mainly focus on sports and mention that social success with the group of young people is also related to the physical aspect” (M3SC25)*.

## Systemic Barriers

Although most respondents felt confident using models of good practice, they noted systemic barriers to good practice, specifically a referral system, and time constraints that did not accommodate obesity treatment, showing that obesity does not fit the system. Additionally, respondents suggested that lack of specialized centers and poor education of healthcare professionals indicated the system doesn't prioritize obesity treatment and prevention.

### Obesity Does Not Fit the System

Respondents frequently noted that clinical consultations should empower families. For this to be achieved, patients needed time to build trust, and healthcare practitioners needed time to adapt to each family's needs; however, time constraints sometimes hindered this. As one respondent described: “*You have to have a lot of time for explanations with these families, (…) you gain their trust this way (.) however doctor's consultation time is shorter than I would need (.) honestly with a patient with obesity, I consume time for 3 consultations”*(F5TC23). Another respondent explained: “*One cannot do a consultation for obesity in 15 or 20 minutes, as for acute pathologies”* (F3TC33).

Healthcare settings and processes were also cited as barriers. One respondent noted that a barrier in seeking treatment for the child's excess weight might be related to the hospital setting:

“*It is a cultural fear of being in a physician's office or in hospital. Parents say: “I don't want to take my child to the hospital because he gets a disease there." Perhaps, de-medicalization of the subject would be a solution, in the sense of not being addressed in a clinical setting as a medical treatment, rather as a behavioral treatment”* (F1TC12).

Some respondents worried that adolescents with risk for morbid obesity were lost in the transition to adult care: “*we lose them there because when they turn 18, they no longer have a trusting vote with us* (pediatricians) …*. They see themselves as adults and as an adult they do not respect what they are told.unfortunately.”* (F3TC33).

### The System Does Not Prioritize Obesity

Several respondents noted that, as opposed to diabetes or cardiovascular diseases, the Romanian medical system does not consider obesity prevention or treatment a priority. As one respondent said: “*Obesity is not seen as a medical condition, it is not seen as a condition that needs treatment. That's why the* (healthcare) *system works poorly here”* (M2SC18). Respondents mentioned lack of training as one example of the deprioritizing of obesity. One pediatrician said: “*I am not very convinced that we, the physicians, are properly educated to understand that obesity is a disease.”* (F2PC7). Another aspect of the deprioritizing of obesity was the delay in referrals, with specialist doctors saying that general practitioners did not address childhood obesity in time:

“*(.) the family physician, or the physicians in schools should have observed that the child has excess weight and they should have addressed it, and get in contact with specialists to receive adequate monitoring. Practically, the system (the medical network for management of obesity) does not exist”* (M2SC18).

Respondents suggested that multiple professionals should be involved in obesity care, including dieticians, psychologists and social services, as needed. However, consultations could rarely be scheduled in 1 day and respondents cited multiple visits as a contributor to families' reduced treatment participation. To increase coordination amongst multiple professionals, respondents underlined the need for specialized centers for childhood obesity treatment, as one respondent noted:

“*Medical barriers are organizational. (.) all consultations should be scheduled and well-coordinated. I'm talking about a consultation with the medical team, followed by a meeting with the dietician and with the psychologist, so that all this can be performed conveniently during one morning. Then the child does not have to miss school once for the doctor appointment, once for the dietician, once for the psychologist”* (F3TC33).

All respondents underlined the need for a systemic approach, with childhood obesity prevention and intervention promoted by government policies, media, kindergartens and schools, family physicians, and general pediatricians.

## Discussion

This is the first study in Romania and in Central/Eastern Europe to investigate the barriers and facilitators healthcare professionals face when communicating with and treating families of children with obesity. The analysis shows that healthcare professionals identified the burden of stigma and the presence of comorbidities as the “tipping points” that lead families to seek help. However, they found that disagreements between family members about the seriousness of obesity, alongside families' distrust of the healthcare system, posed barriers to treatment. To build trust and engage families in treatment, the participating healthcare professionals followed models of good practice, consisting of a gentle approach and patient-centered care. Yet the respondents argued that treatment was often limited due to systematic barriers, including the referral system and time constraints, as well as the lack of specialized centers and poor education of healthcare professional. These were cited as examples of the healthcare system's deprioritizing of childhood obesity treatment and prevention.

The respondents' observation that children's experiences of weight stigma were the main motivation for treatment seeking suggests that treatment was initiated too late, and that children starting treatment had already experienced emotional and social hurt related to obesity. Children and adolescents with obesity who experience stigma suffer from psychological, physical, and behavioral difficulties that increase the risk of social isolation and weight gain (19). In addition, experiences of stigma have detrimental effects on healthy eating and engaging in physical activity (20–22), access to treatment (23), and adherence to weight loss related treatment (21, 24), suggesting that children who begin treatment after having experienced stigma might not fully benefit from it.

The respondents characterized childhood obesity an “invisible disease,” with family members seeking treatment only after observing comorbid conditions. Delayed treatment seeking, until childhood obesity becomes severe or complicated by other diseases, has been observed in other studies (25–28). Moreover, in a United States based study, Eli et al. (29) found that parents of young children believe that obesity becomes a problem only at the beginning of primary school, when children might face bullying, or when comorbidities occur.

To make the “invisible” nature of excess weight concrete, the healthcare professionals in our study reported that they used growth charts to objectively compare the child's weight status to with a healthy weight reference population. Growth charts are useful and acceptable tools that professionals commonly use to improve counseling and facilitate behavior change (30). Therefore, these charts should be shown to parents before children develop obesity, to assist early prevention efforts. The recent study by Sjunnestrand et al. conveys the importance of teaching parents to understand child growth charts early on, and thus prevent obesity and associated comorbidities (9).

Several layers of distrust within the family and between the family and the medical practitioners were noted in the interviews. Within the family, respondents said that often only one parent or some family members recognized the excess weight, while others did not, leading to disagreements on how to help the child. Similar findings were reported in an Australian interview study by Jackson et al. (31), who found that, in almost half of the families, fathers either did not recognize the child's excess weight or were in denial about it. The authors therefore proposed addressing children's excess weight as a familial issue, rather than as an individual's problem, in order to get the family to work together (31). Between the family and the medical practitioners, tension arose around definitions of childhood obesity. Moreover, respondents underscored that families were reluctant to participate in clinical trials for obesity treatment, suggesting distrusts of the healthcare system. This reluctance, according to the respondents, could indicate fear of being experimented on. At the time of publication there were no studies in Romania investigating communication between healthcare practitioners and families of children with obesity in relation to clinical studies. However, studies investigating patient's satisfaction with the Romanian healthcare system, showed high levels of dissatisfaction, and lack of trust in medical services and healthcare professionals (32, 33). Patient dissatisfaction was related to communication barriers with healthcare professionals, as well as low quality accommodation, food and hygiene in hospitals, which thus failed to provide patients with a sense of security (32–34). Distrust toward medical services might lead to distrust toward medical studies. The local STOP project team in Romania has observed that recruitment to the study (35) has been limited. Similar concerns were reported in a US-based study which evaluate clinical trial acceptance (36). It has been suggested that addressing patients' fears with detailed explanations of methodology and patient safety measures could improve recruitment. Davidson and Vigden have recently evaluated the acceptance of participation in childhood obesity studies in Australia (37). They found that parents' decision to enroll in obesity programs was influenced by experiences with previous attempts to lose weight and their child's emotional state (37).

In our study, tension between families and healthcare professionals created barriers in communication. This is in line with a meta-synthesis of qualitative studies showing that different perceptions of excess weight between families and doctors are common barriers (12). These barriers, unless understood and addressed, might limit treatment success (38). In this study, the respondents addressed these barriers by using models of good practice when communicating with families about children's excess weight. A gentle approach, which increased parental awareness about childhood obesity while avoiding offense, was key. Previous research has shown the importance of avoiding judgment in developing successful communication with families of preschoolers with obesity (39). However, clinical practice alone does not improve communication skills, and when communication skills are lacking, practitioners usually avoid difficult conversations (38). Therefore, formal training is crucial in improving communication with families (40). In the present study, the respondents identified lack of training as a barrier to communicating with families. This barrier should be addressed by governmental policies to improve clinical continuing education.

Models of good practice reported by the respondents also included a patient centered care approach. Practitioners adapted treatment recommendations to the possibilities and needs of patients and families. Promotion of informed choice as part of the patient centered care approach has been emphasized in different medical fields (41, 42). It includes a stepwise approach culminating with patients expressing their own choice, after the healthcare professional has presented different treatment pathways with their pros and cons (41, 42).

A key finding was that respondents identified changes in the healthcare system as crucial to improving capacity for childhood obesity interventions. They noted that primary care physicians were often slow to refer families, and that families initiated treatment in secondary and tertiary care, only after comorbidities were observed. If obesity treatment were promoted in primary care, excess weight could be addressed earlier, with benefits to both patients and the healthcare system, as shown by a recent guideline issued by World Health Organization (43). Interestingly, while Romania and Sweden have different healthcare systems, Sjunnestrand et al. also reported that healthcare practitioners felt systematic changes were needed to improve treatment referral and uptake (9). Additionally, because childhood obesity treatment in Romania is associated with secondary and tertiary care, the respondents noted that families might refuse or postpone obesity treatment due to fears related to hospital settings. Offering a community-based, multi-disciplinary setting for childhood obesity treatment might increase the acceptability of treatment. The healthcare system could also benefit by limiting overcrowding in secondary and tertiary care and encouraging earlier treatment initiation (44).

### Strengths and Limitations

This is the first study to investigate Romanian healthcare professionals' perspectives on communicating with families about childhood obesity. The study used the interview guide developed by Sjunnestrand et al. (9), who interviewed a homogenous group of pediatric nurses working in primary care centers in Sweden. In the present study, the interviews were conducted with a heterogeneous group of healthcare professionals from primary, secondary and tertiary care, in order to present various perspectives on communication with families of children with excess weight in Romania. Thus, while the study benefited from using an established interview guide, the different categories of healthcare professionals interviewed precluded a meaningful comparison with the Swedish dataset. A formal list of pediatric dieticians was not available, as at the time of the study a national association of dieticians did not exist. Therefore, we invited the authors' personal contacts to participate, and we recognize this may have limited the diversity of dietician participants. The dieticians invited could not provide contact for other colleagues involved pediatric nutrition, possibly because of low numbers or lack of formal association. Future research involving dieticians would be strengthened by the use of snowball sampling, which could provide a more diverse sample. The study was potentially limited by selection bias, since participants who agreed to be interviewed might have had a particular interest in childhood obesity. The study was also limited by the low response rate, likely due to the interview timeframe, which overlapped with the COVID-19 pandemic lockdown.

### Future Directions

While this study has focused on healthcare professionals' perceptions of communicating about and treating childhood obesity, it would be important to understand families' experiences, as well. We plan to investigate the experiences of parents and children as part of the More and Less Study Europe (35). The study's findings convey the importance of establishing a professional network in Romania for the management of childhood obesity. Moreover, services provided by dieticians should be included in the Romanian Health Insurance network, to facilitate the integration of dietetics service into standard childhood obesity treatment. In addition, the primary medical care in kindergarten and schools in Romania, might be used, in a step-by-step approach, for raising awareness about childhood obesity, reducing obesity stigma, and encouraging treatment initiation.

## Conclusions

The interviewed doctors and dieticians in Romania identified patient-centered care as key to treating children with obesity and building trust with their caregivers. However, their efforts are hindered by healthcare system barriers, including the lack of specialized centers, training and a referral system. The findings therefore suggest that, to improve childhood obesity prevention and treatment, systematic barriers should be addressed.

## Data Availability Statement

The original contributions presented in the study are included in the article/Supplementary Material, further inquiries can be directed to the corresponding authors.

## Ethics Statement

The studies involving human participants were reviewed and approved by Scientific Research Ethics Committee Board of the Victor Babes University of Medicine and Pharmacy Timisoara (no.06/02.03.2020). The patients/participants provided their written informed consent to participate in this study.

## Author Contributions

AC-E, PN, and AE developed the interview guide. SP conducted the interviews. AC-E and CLS wrote the first draft of the manuscript under supervision of PN. KE contributed to the analysis and to the writing of the manuscript. All authors contributed to final editing of the document and approved of the last version of the manuscript.

## Conflict of Interest

The authors declare that the research was conducted in the absence of any commercial or financial relationships that could be construed as a potential conflict of interest.

## Supplementary Material

Supplement 1 COREQ checklist. Includes the COREQ checklist for this submission.

## Abbreviations

COREQ: COnsolidated criteria for REporting Qualitative research
COSI: European Childhood Obesity Surveillance Initiative
F: female
M: male
PC: primary care
SC: secondary care
STOP: Science and Technology in childhood Obesity Policy
TC: tertiary care.
